# Suggestions for the decision making in subjective cognitive complaints

**DOI:** 10.1007/s40520-024-02875-8

**Published:** 2025-01-21

**Authors:** Benedetta Basagni, Eduardo Navarrete

**Affiliations:** 1 Clinica di Riabilitazione Toscana, CRT, Montevarchi, Arezzo, Italy; 2 Dipartimento di Psicologia dello Sviluppo e della Socializzazione, Università di Padova, Padova, Italy

**Keywords:** Subjective Cognitive Complaints (SCC), Dementia, Anamnesis, Neuropsychological Assessment, Risk Factors

## Abstract

In recent years, the increasing life expectancy has underscored the importance of cognitive health alongside physical well-being, particularly because healthy adults may report subjective cognitive complaints (SCC), often related to memory. These complaints may or may not align with objective cognitive impairments, fueling ongoing debates about whether SCC could serve as an early indicator of dementia. While some studies suggest SCC as a potential precursor to dementia, others propose that these complaints may merely co-occur with cognitive decline. Despite the lack of consensus, addressing SCC remains crucial for early intervention, especially as emerging treatments for dementia show promise when applied at early stages. Risk factors associated with dementia, such as age, education, family history, and comorbid conditions like depression and diabetes, have been incorporated into predictive models. However, clinical practice continues to rely heavily on neuropsychological assessments to bridge subjective complaints with objective cognitive performance and may often require additional investigations, such as neuroimaging. Factors such as cognitive reserve, depression, stress, sleep disturbances, and personality traits also play significant roles in the interpretation of SCC. Some of these conditions may potentially mask underlying cognitive decline. A comprehensive clinical evaluation, integrating neuropsychological testing with a thorough anamnesis, can help distinguish between cognitive disorders and other contributing factors. Here, we propose a flowchart to guide clinicians in the management of SCC, integrating key factors to enhance diagnostic accuracy and inform treatment decisions. Despite the challenges involved, a careful and holistic approach remains essential for effective patient care.

## Introduction

A major debate in the scientific literature centers on whether SCC can be considered as an early indicator of dementia’s prodrome. Mendonca’s [1] systematic review highlights the risk of cognitive impairment in individuals with SCC, emphasizing a link between self-recognition of cognitive inefficiency and a significantly elevated risk of progressing to dementia. Recently, network analysis has been used to explore the relationship between SCC and normal versus pathological aging [2], suggesting that SCC might represent an intermediate stage between normal aging and mild cognitive impairment. However, these findings are not universally accepted. Some longitudinal studies report that individuals with more initial subjective memory complaints did not experience a faster decline in objective cognitive performance, suggesting that subjective cognitive impairments may not predict cognitive decline but rather co-occur with it [3].

Nevertheless, addressing these subjective disorders presents a significant challenge for clinicians, especially given the current development of innovative dementia treatments that seem more effective when administered in the early stages (see Ebell et al., [4] for a review).

To promptly initiate patient care, considerable efforts have been made to identify risk factors for dementia development. Biomarker analysis, including APOE gene, represents a promising avenue of research with the potential to facilitate early detection of cognitive decline (see Budelier et al., [5] for a review). Despite its potential, this approach has yet to be fully integrated into clinical practice, and there is still a need to establish clear criteria for laboratory analysis access. As a result, the assessment of behavioral patterns and risk factors remains essential for clinicians performing initial cognitive screenings.

A comprehensive neuropsychological examination is a critical initial step for determining the need for further investigation. It aligns the patient’s self-reported experiences with objective data, highlighting deviations from expected performance norms. When objective performance deficits emerge during these assessments, further investigations, including brain scans to rule out other neurological causes and to further assess brain functioning, are generally recommended.

However, beyond neuropsychological test outcomes, other factors may influence cognitive performance and subjective experiences of individuals with SCC. A comprehensive anamnesis, accounting for all factors influencing the individual’s perception of cognitive inefficiency, is essential. Informed decision-making, such as whether to prescribe additional tests or simply monitor the profile, relies on integrating neuropsychological assessment results with clinical and behavioral data.

In a recent predictive model by Anatürk and colleagues [6], factors forecasting dementia development over 14 years were identified: advanced age, lower educational attainment, diabetes, depression, stroke history, family history of dementia, socioeconomic disadvantage, hypertension, cholesterol status, male gender, and household occupancy. By studying a large sample of individuals, the authors developed a very useful program to calculate an individual’s dementia risk and classify healthy subjects into low-risk and high-risk groups. Nevertheless, the management of patients with SCC remains complex and challenging.

For example, how should clinicians interpret a clinical scenario where significant subjective cognitive disturbances are not matched by poor performance on neuropsychological tests? Can the absence of abnormalities in cognitive screening alone justify discontinuing ongoing monitoring of the cognitive profile? What specific anamnesis data can help clinicians consider alternative diagnoses beyond cognitive deterioration?

The Comprehensive Geriatric Assessment [7] is a valuable tool for supporting the diagnostic process. This multidimensional and multidisciplinary assessment focuses on elderly patients and encompasses three dimensions: physical performance, functional ability, and cognition and mental health. Additionally, Devita and collaborators [8] recently emphasized the importance of including a measure of cognitive reserve (CR) when suspecting cognitive impairment. CR refers to the accumulation of stimulating experiences throughout an individual’s life, including education, work, and recreational activities. Evidence shows that CR may act as a protective factor against neurodegenerative diseases (e.g., Stern et al., [9]). Interestingly, a high level of CR might initially mask objective cognitive deterioration. In individuals with high CR, cognitive decline may remain ‘silent’ due to the greater availability of neurological substrates and redundant networks in the brain. Therefore, subjective cognitive disturbances should be considered with greater caution in these cases, as individuals may become aware of inefficiencies before they are overtly apparent. Interesting, patients with high CR are also more likely to report inefficiencies compared to those with lower cognitive reserve, due to their greater awareness and perceived sense of self-efficacy. As a result, individuals with high CR require vigilant monitoring.

Nonetheless, CR is not the only factor to consider when addressing SCC. In 2013 [10], Mark and colleagues sought to standardize guidelines for evaluating SCC, identifying specific “red flags” that warrant further investigation in patients who report subjective cognitive deficits but show no abnormalities on brief cognitive screening. They highlight certain anamnesis elements that merit attention, such as high education and socioeconomic status, distinct complaints, marital status, age under 80, decline in instrumental activities of daily living, adequate effort on neuropsychological tests, and concurrent risk factors like depression, vascular or neurological diseases, or a slow gait. The authors recommend conducting a comprehensive neuropsychological evaluation and considering neuroimaging studies and biomarker assessments when most of these conditions are present.

This “*Perspective*” aims to prompt further reflection on the aspects to investigate when dealing with a subject exhibiting SCC. We will first outline the aspects to consider and then present a potential flowchart to aid in understanding the manifestation of SCC.

## Critical factors analysis in the management of subjective cognitive disorder

*Depression and Stress.* Existing literature highlights a significant association between subjective memory disturbances and depressive symptoms, particularly among older individuals. The cognitive decline associated with mood disorders is sometimes referred to as ‘depressive pseudodementia’. According to the DSM-V, individuals with major depression often report impaired cognitive abilities, difficulty concentrating, making minor decisions, and performing cognitively demanding tasks. When the depressive episode is effectively treated, the associated memory problems generally resolve. It is noteworthy that distinguishing between the symptoms of organic dementia and those stemming from a depressive state can be difficult, as they frequently coexist and influence each other.

Perceived stress is also considered a potential predictor of SCC. During the medical history interview, it is crucial to inquire about stressful life events such as illness, the loss of a loved one, or financial issues. In summary, a thorough clinical interview, potentially supplemented by a depression and anxiety screening questionnaire, is strongly recommended for evaluating SCC.

*Personality.* Research on personality traits has identified links between neuroticism and pronounced conscientiousness with SCC. Individuals with rigid and highly conscientious personalities may be more likely to perceive cognitive changes as pathological, as they may struggle to accept normal age-related cognitive changes. While comprehensive personality assessments through specific tests may not always be practical, a clinical interview can provide valuable insights into the individual’s personality profile.

*Sleep Quality.* Chronic sleep disturbances can negatively impact cognitive function, and evidence suggests a higher likelihood of subjective memory decline in middle-aged and older adults with insomnia disorders. Assessing sleep quality is an essential aspect of evaluating SCC.

*Familiarity with Neurodegenerative Pathology.* Certain forms of dementia may have a familial predisposition, and in some rare cases, a strong genetic link. Family history is a routine component of neurological assessments. Additionally, a family history of neurodegenerative diseases can trigger anxiety and concerns about developing similar conditions, which may lead individuals to perceive cognitive inefficiencies even in the absence of objective deficits. Therefore, it is important to inquire not only about dementia cases in the patient’s family but also about the patient’s engagement and personal experiences related to these events. Direct questions regarding the patient’s concerns about developing the same pathology can also be valuable.

*Drugs and Psychostimulants.* Certain substances, including anticonvulsants, benzodiazepines, and alcohol, are known to negatively impact cognitive functions. Long-term studies and brain imaging have consistently shown that excessive alcohol consumption increases the risk of cognitive impairment. Additionally, benzodiazepine abuse can notably affect the speed of information processing. Thus, a thorough inquiry into the use or misuse of psychoactive substances should be a key component of the medical history in SCC.

*Menopause.* For females aged 45 to 55, gathering information on the progression of menopause is important. Studies indicate that cognitive impairments often escalate during the menopausal transition, with declines in attention, working memory, and reductions in medial temporal lobe volume. Additionally, women undergoing estrogen-lowering treatments experience greater cognitive impairment, particularly in working memory and executive function.

*Chronic pain*. Cognitive impairment is frequently associated with chronic pain (lasting more than three months), especially in the elderly population. The scientific literature most commonly reports neuropsychological disturbances such as impaired attention, memory, executive function, and overall cognitive performance. Furthermore, patients with chronic pain often rely on large quantities of pharmacological treatments, including opioids, which can have additive effects on cognitive efficiency.

*Self/Other Evaluation.* Individuals whose subjective complaints are corroborated by objective validation from a family member are at a higher risk of progressing to dementia. The confirmation of impaired cognitive performance by an informant can be considered an additional risk factor. Compared to cases based solely on self-reported impairments, instances where cognitive deficits are confirmed by an informant have a higher risk of progressing to mild cognitive impairment or dementia. While not all patients may have family members present during their evaluation, it is beneficial to inquire whether the individual reporting cognitive deficits has discussed their observations with a family member.

### Flowchart

**Fig. 1:**
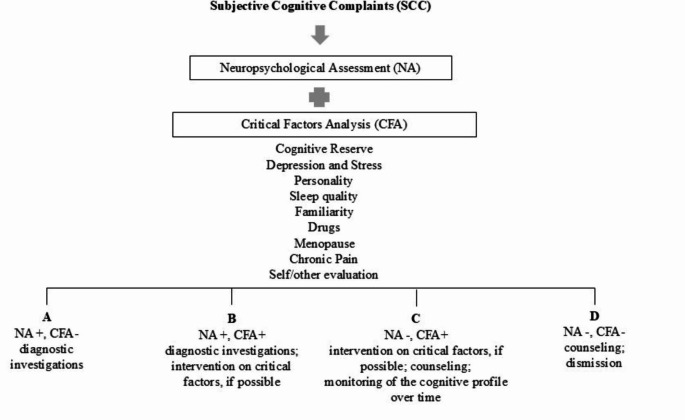
Flowchart: Recommendations to interpret SCC. Notes: NA+, cognitive impairment in any domain; NA-, normal neuropsychological examination; CFA+, critical factors analysis that may account for SCC; CFS-, critical factors analysis that do not account for SCC

Figure 1 illustrates a framework for managing SCC.

The initial step involves conducting a Neuropsychological Assessment (NA) to identify any deviations from normal cognitive function. This assessment should include not only a general dementia screening test, such as the internationally known MMSE, MoCA, or ACE-III, but also specific evaluations of verbal memory, attention, and executive functions, utilizing tools that are available to the neuropsychologists and standardized for each language. Establishing a baseline through the NA is crucial, as it provides a key reference point for tracking changes in cognitive performance over time.

The NA should be complemented by a thorough analysis of critical factors (CFA) related to SCC. Some of these factors may offer alternative explanations for cognitive impairment, such as substance abuse, sleep deprivation, menopause, chronic pain, and personality traits. Others, including family history and depression, can prompt individuals to seek help and represent actual risk factors. As previously mentioned, high CR may lead individuals to express concerns more readily but can also mask underlying cognitive disorders, potentially resulting in a well-conducted NA even if early cognitive decline is present. Additionally, if SCC is supported by an informant’s evaluation indicating worsening performance, this should prompt the need for more in-depth diagnostic investigations. Based on the assessments from the NA and the CFA, four potential decision-making options can be derived.

- If the NA reveals deficient scores and no critical factors are identified, it is essential to continue the diagnostic process (e.g., neuroimaging and biomarker analysis).
- If the NA is positive and the CFA suggests alternative hypotheses, intervention should address these factors concurrently (e.g., treating depression or a sleep disorder if present) alongside with the continuation of the diagnostic process (e.g., neuroimaging and biomarker analysis).
- If the NA is negative but risk factors or other elements suggest that the subjective disorder may be caused by other issues, the clinician should focus on these areas and monitor the cognitive profile over time.
- If the NA is negative and no other factors justify the disturbance, if the SCC is not supported by family observations, and if predisposing factors (e.g., high conscientious personality) are present, diagnostic conclusions might suggest that the changes are due to aging and personal sensitivity to the issue. In such cases, a counseling intervention could be beneficial for understanding the underlying mechanisms of perceived inefficiency and explaining the genesis of the problem.

## Conclusions

In summary, evaluating SCC is a complex task that requires integrating neuropsychological test results with a thorough examination of the factors influencing the subjective experience. Many of these factors play dual roles, acting both as risk factors and potential confounders, representing two aspects of the same issue. For example, depression, cognitive reserve, and family history can affect individuals reporting cognitive inefficiencies by both predisposing them to neurodegenerative pathology and potentially leading to inaccurate self-assessments of their cognitive abilities.

From this *perspective*, we have outlined a potential process to support clinicians in the diagnostic process. This framework serves two main purposes: it aids in formulating a diagnostic hypothesis and provides a qualitative assessment of the risk associated with dementia development, as well as the need for ongoing cognitive monitoring. Despite the inherent risk of false positives and false negatives due to the complexity of interacting and confounding factors, this approach can enhance both reflection and decision-making.
